# Survival Strategies of Pathogenic *Candida* Species in Human Blood Show Independent and Specific Adaptations

**DOI:** 10.1128/mBio.02435-20

**Published:** 2020-10-06

**Authors:** Philipp Kämmer, Sylvie McNamara, Thomas Wolf, Theresia Conrad, Stefanie Allert, Franziska Gerwien, Kerstin Hünniger, Oliver Kurzai, Reinhard Guthke, Bernhard Hube, Jörg Linde, Sascha Brunke

**Affiliations:** a Department of Microbial Pathogenicity Mechanisms, Leibniz Institute for Natural Product Research and Infection Biology—Hans Knöll Institute, Jena, Germany; b Research Group Systems Biology and Bioinformatics, Leibniz Institute for Natural Product Research and Infection Biology—Hans Knöll Institute, Jena, Germany; c Research Group Host Fungal Interfaces, Leibniz Institute for Natural Product Research and Infection Biology—Hans Knöll Institute, Jena, Germany; d Research Group Fungal Septomics, Leibniz Institute for Natural Product Research and Infection Biology—Hans Knöll Institute, Jena, Germany; e Institute for Hygiene and Microbiology, University of Würzburg, Würzburg, Germany; f Friedrich Schiller University Jena, Jena, Germany; g Center for Sepsis Control and Care, Jena University Hospital, Jena, Germany; h Research Group PiDOMICS, Leibniz Institute for Natural Product Research and Infection Biology—Hans Knöll Institute, Jena, Germany; i Institute for Bacterial Infections and Zoonoses, Federal Research Institute for Animal Health—Friedrich Loeffler Institute, Jena, Germany; Tel Aviv University

**Keywords:** *Candida albicans*, *Candida glabrata*, *Candida parapsilosis*, *Candida tropicalis*, pathogen evolution, dual-species RNA-seq, host-pathogen interactions

## Abstract

Only four species, Candida albicans, C. glabrata, C. parapsilosis, and C. tropicalis, together account for about 90% of all *Candida* bloodstream infections and are among the most common causes of invasive fungal infections of humans. However, virulence potential varies among these species, and the phylogenetic tree reveals that their pathogenicity may have emerged several times independently during evolution. We therefore tested these four species in a human whole-blood infection model to determine, via comprehensive dual-species RNA-sequencing analyses, which fungal infection strategies are conserved and which are recent evolutionary developments. The *ex vivo* infection progressed from initial immune cell interactions to nearly complete killing of all fungal cells. During the course of infection, we characterized important parameters of pathogen-host interactions, such as fungal survival, types of interacting immune cells, and cytokine release. On the transcriptional level, we obtained a predominantly uniform and species-independent human response governed by a strong upregulation of proinflammatory processes, which was downregulated at later time points after most of the fungal cells were killed. In stark contrast, we observed that the different fungal species pursued predominantly individual strategies and showed significantly different global transcriptome patterns. Among other findings, our functional analyses revealed that the fungal species relied on different metabolic pathways and virulence factors to survive the host-imposed stress. These data show that adaptation of *Candida* species as a response to the host is not a phylogenetic trait, but rather has likely evolved independently as a prerequisite to cause human infections.

## Abstract (precis)

To ensure their survival, pathogens have to adapt immediately to new environments in their hosts, for example, during the transition from the gut to the bloodstream. Here, we investigated the basis of this adaptation in a group of fungal species which are among the most common causes of hospital-acquired infections, the *Candida* species. On the basis of a human whole-blood infection model, we studied which genes and processes are active over the course of an infection in both the host and four different *Candida* pathogens. Remarkably, we found that, while the human host response during the early phase of infection is predominantly uniform, the pathogens pursue largely individual strategies and each one regulates genes involved in largely disparate processes in the blood. Our results reveal that C. albicans, C. glabrata, C. parapsilosis, and C. tropicalis all have developed individual strategies for survival in the host. This indicates that their pathogenicity in humans has evolved several times independently and that genes which are central for survival in the host for one species may be irrelevant in another.

## INTRODUCTION

Bloodstream infections can lead to sepsis, a major public health concern with high mortality rates caused by a dysregulated systemic inflammatory immune response (1–3). While cases of sepsis are mostly bacterial in origin, fungi can cause sepsis also (4), and Candida albicans, C. glabrata, C. parapsilosis, and C. tropicalis together account for at least 90% of all fungal bloodstream infections (5–7).

Apart from C. glabrata, these pathogens are found in the CTG clade of fungi, sharing a unique difference with respect to codon translation. This suggests that their pathogenicity strategies evolved at the base of this phylogenetic branch, while the presence of nonpathogenic species interspersed with the pathogens (8, 9) rather suggests independent evolutionary origins. Finally, comparative genomic analyses suggest that certain lineages develop pathogenicity due to previous adaptations to the host or the environment. It remains unclear whether these fungi generally follow similar infection and survival strategies in the host due to their relatedness (10)—an issue which we address in this work.

The pathogenic *Candida* species are usually commensals which colonize skin or mucosae without causing clinical symptoms, but in patients with immunodeficiencies or damaged anatomical barriers, dissemination into the bloodstream can occur (11, 12). Clinical differences are known among the species. While C. glabrata has a high incidence in the elderly, C. parapsilosis causes high mortality in low-birth-weight neonates (13, 14). C. tropicalis is often associated with neutropenia or malignancy (15, 16). Overall, C. albicans remains the most prevalent cause of invasive candidiasis, but the frequency of other *Candida* species has increased to about 50% (11, 17, 18). Notably, non-*albicans Candida* species, in particular, C. glabrata, are often more resistant to common antifungals (19, 20).

For their dissemination, *Candida* cells must enter the bloodstream, where they face an entirely new, harsh environment. Access to nutrients is strictly limited, and the innate immune system combats invading pathogens immediately. Monocytes and, particularly, neutrophils act as the first line of cellular defense in the bloodstream (21). Consequently, neutropenia is associated with poor prognosis in candidemic patients (22) and neutrophils govern the transcriptional response of C. albicans in the blood (23, 24). It is unknown whether these observations also apply to pathogenesis of other *Candida* species. In addition, the lack of differentiable clinical symptoms complicates the identification of infecting *Candida* species. Detection of distinct patterns in the host or fungal response therefore has the potential to both improve understanding of the pathobiology of *Candida* and reveal species-specific biomarkers.

Models to investigate clinical events in the laboratory have various limitations. *In vitro* infection models of primary immune cells have helped identify important virulence factors of *Candida* species (25–30) but lack the complex interplay present among different components of the immune response. Animal *in vivo* models, mainly mouse models, give a better understanding of the onset and progression of disseminated candidiasis (27, 31–38). However, most *Candida* species are normally not commensals or pathogens of these model hosts (39) and their immune system differs in important aspects from that of humans (reviewed in reference 40). The use of human whole blood *ex vivo* can overcome some of these limitations (41). Our own previous studies explored the transcriptional responses of C. albicans or host during blood infections (23, 42–44) and characterized the interplay of innate immune cells and blood components with C. albicans or C. glabrata (24, 45–47).

Here, we employed a complex, time-resolved *ex vivo* whole-blood infection model which mimics the early dissemination stage of candidemia (23, 24) to investigate (i) molecular and cellular events during infection and (ii) the interdependent transcriptional patterns of human host and common *Candida* species by dual-species RNA-sequencing (RNA-seq). We show that the human host responds to the challenge from *Candida* spp. with a predominantly uniform and strong proinflammatory cytokine response, while the fungal responses are dominated by species-specific adaptations, indicating that their pathogenicity evolved independently. These findings are supported by the fact that deletions of orthologous genes have different impacts on the survival of C. albicans and C. glabrata.

(A previous version of this manuscript is part of the dissertation of Theresia Conrad, submitted to the Friedrich Schiller University Jena.)

## RESULTS

### Mimicking *Candida* bloodstream infections *ex vivo*.

Dissemination via the bloodstream is a hallmark of invasive *Candida* infections (45, 48). We applied an improved *ex vivo* whole-blood infection model (originally described in reference 24) to simulate early dissemination stages of *Candida* species, using an infection dose (10^6^ cells/ml) that was up to 50-fold lower than those used in previous studies (23, 42, 43).

**FIG 1:**
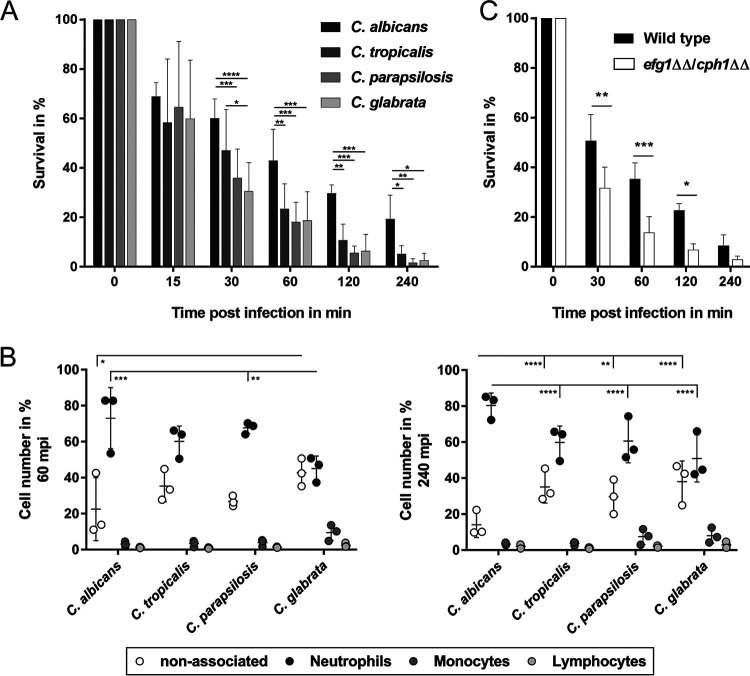
*Candida* species interact with human immune cells and are killed immediately upon blood exposure. (A) Within 1 h of blood infection, the majority of fungal cells were killed. Although the killing kinetics between the fungal species were similar, C. albicans survived to an overall larger extent. (B) By 60 mpi, the majority of fungal cells were already associated with immune cells of human whole blood, predominantly with neutrophils. C. glabrata and C. parapsilosis were associated with monocytes in a larger amount than C. tropicalis and C. albicans 240 mpi. (C) The afilamentous C. albicans *efg1*ΔΔ *cph1*ΔΔ mutant was killed faster than the wild type, similarly to the non-*albicans Candida* species. Data show means of results from four (A) or three (B and C) independent experiments from different donors ± SD. *, *P* value < 0.05; **, *P* value < 0.01; **, *P* value < 0.001; ****, *P* value < 0.0001 (2-way ANOVA).

We found for all species that a substantial part of the population had been killed within 30 min postinfection (mpi), demonstrating the high antifungal activity of healthy human blood (Fig. 1A). Within 60 mpi, the level of killing was about 80%, with the notable exception of C. albicans (57.3%). This continued for up to 4 h with significantly more surviving C. albicans fungi (19.1%) than C. tropicalis, C. parapsilosis, or C. glabrata fungi (5.2%, 1.7%, and 2.7%, respectively). Immediately after entering the bloodstream, pathogens encounter cells of the innate immune system. Leukocytes interacted rapidly with *Candida* cells in human blood; at 60 mpi (Fig. 1B), the vast majority of the fungal cells were predominantly in contact with neutrophils (45.1% to 73.1%) and a much smaller proportion with monocytes (3.1% to 9.5%). Species-specific differences were observed at both early and late time points; C. albicans was frequently associated with neutrophils at 240 mpi (80% versus 50% to 60% for the other species) and was only rarely found not to be associated with any immune cell (14.1% versus 29.7% to 38%). C. glabrata interacted much more avidly with monocytes than the other species did starting at 60 mpi (9.6% versus 3.1 to 3.9%), in agreement with previous results (45, 47). At 240 mpi, C. parapsilosis was also more frequently associated with monocytes than C. albicans and C. tropicalis were (7.5% versus 3.4% to 3.7%). Flow cytometry-based data were qualitatively validated by blood smears, and contact with blood cells was observed microscopically (see Fig. S1A in the supplemental material). Only C. albicans formed filaments starting at 60 mpi. We therefore investigated whether this ability of C. albicans to form hyphae contributes directly to its survival in blood. An afilamentous mutant, *efg1*ΔΔ *cph1*ΔΔ, was killed significantly faster than the wild type (Fig. 1C), and the survival rate was reduced to 2.9% at 4 h. The mutant is defective in hyphal morphogenesis and transcription of hypha-associated genes (49), which indicates that these processes are important contributors to C. albicans survival in human whole blood.

10.1128/mBio.02435-20.1 FIG S1 *Candida* cells associate with immune cells and activate neutrophils during blood infection. (A) Blood smears of *Candida* species blood infections displayed the interaction of host and fungal cells. C. albicans changes morphology by forming germ tubes 60 mpi and filaments 120 mpi. Morphological alterations were not observed for C. tropicalis, C. parapsilosis, and C. glabrata. Scale bar: 10 μm. (B) Levels of the PMN activation markers CD69, CD66b, and CD11b increased and that of CD16 decreased upon *Candida* blood infection compared to mock infection. Download FIG S1, TIF file, 2.7 MB. Copyright © 2020 Kämmer et al. 2020 Kämmer et al. This content is distributed under the terms of the Creative Commons Attribution 4.0 International license.

As *Candida* cells interacted predominantly with neutrophils in blood, we determined levels of neutrophil activation. Surface levels of the general early activation marker CD69 (activation inducer molecule [AIM]) were slightly elevated at 240 mpi in the presence of all four *Candida* species, with C. glabrata inducing the largest increase (Fig. S1B). Levels of CD11b (CR3A/ITGAM [integrin alpha M]), mediating leukocyte adhesion, and the degranulation marker CD66b (CEACAM8) were also increased compared to the mock-infection results, with significantly elevated CD66b levels seen upon C. albicans infection. In contrast, the levels of CD16 (FcγRIII) decreased under all four fungal infection conditions. These results demonstrate robust neutrophil activation by all four species.

### The human transcriptional response is mainly species independent.

Having characterized the overall pattern of interaction of *Candida* species with blood-borne immune cells, we next monitored the global transcriptional response of host and fungal cells in a kinetic, dual-species RNA-seq approach.

**FIG 2:**
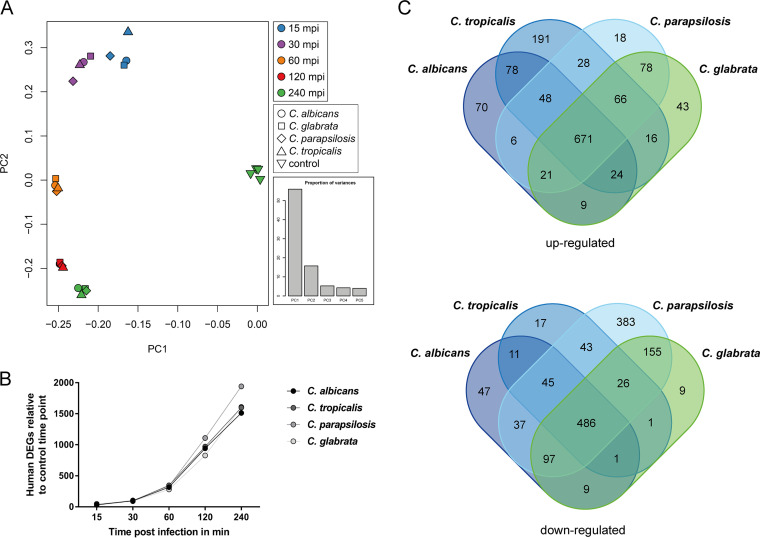

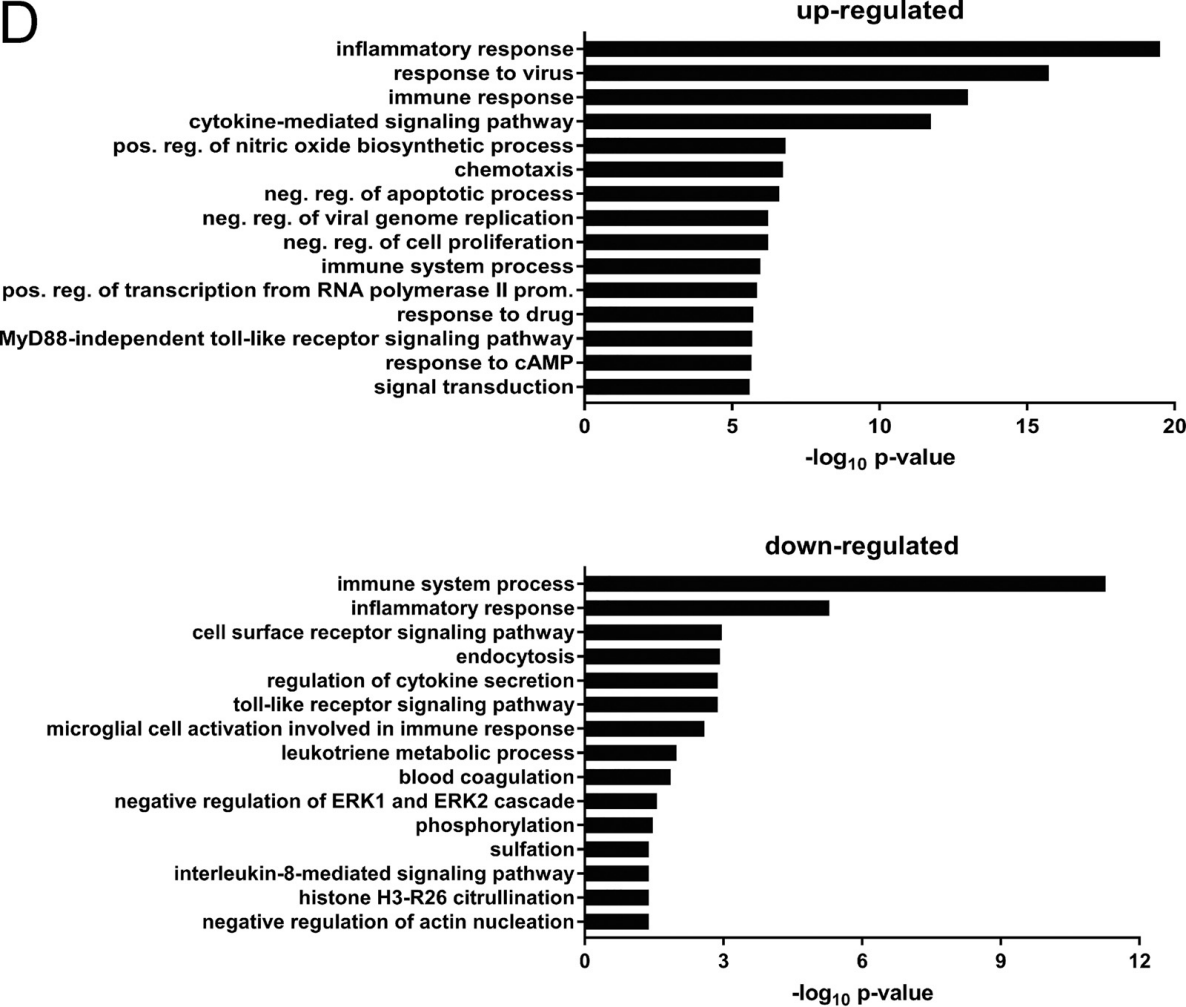
*Candida* species induce a mainly species-independent human transcriptional core response. (A) Principal-component analyses (PCA) revealed higher similarity between samples of one time point (same color) than one species (same icon). Mock-infected control samples (▽) were clustered together and clearly separated from all infection samples. (B) The transcriptional host response started in a restrained manner with only a few regulated genes seen 15 mpi but distinctly increased during the time course of infection with similar kinetics between the four *Candida* infections. (C) Venn diagrams illustrate that in response to *Candida* blood infections, about 670 and 490 human genes were commonly up- and downregulated, respectively. (D) Functional gene ontology (GO) analyses were performed to identify enriched biological processes of common up- or downregulated human genes. Immune system processes such as inflammatory response or cytokine-mediated signaling (both upregulated) or Toll-like receptor signaling (downregulated) governed the human core response, indicating a strong but balanced response to *Candida* blood infections. ERK, extracellular signal-regulated kinase.

A global overview of the human samples by principal-component analyses (PCA) showed that the time point postinfection rather than the infecting species governs transcriptional variance, which clearly differed from the results seen with noninfected samples (Fig. 2A). This human host response was characterized by a rapid increase in the number of regulated genes, from only 30 to 50 at 15 mpi to a maximum of 1,940 differentially expressed genes (DEGs) at 240 mpi during C. parapsilosis infection (Fig. 2B), with similar numbers determined for the other species (see Table S2 in the supplemental material).

By comparing host transcriptional changes over all time points (Fig. 2C), we found a common core response to fungal infections of 671 up- and 486 downregulated “quadruple” genes, differentially regulated at least once in all four infection kinetics. Smaller numbers of genes were differentially expressed in a species-specific manner, from a maximum of 383 DEGs downregulated for C. parapsilosis to 9 DEGs downregulated only in response to C. glabrata. In summary, the human transcriptional response to infecting *Candida* species is predominantly uniform within the first 4 h postinfection, with only a few detectable instances of unique regulation.

### Immune system processes govern the human transcriptional response.

**FIG 3:**
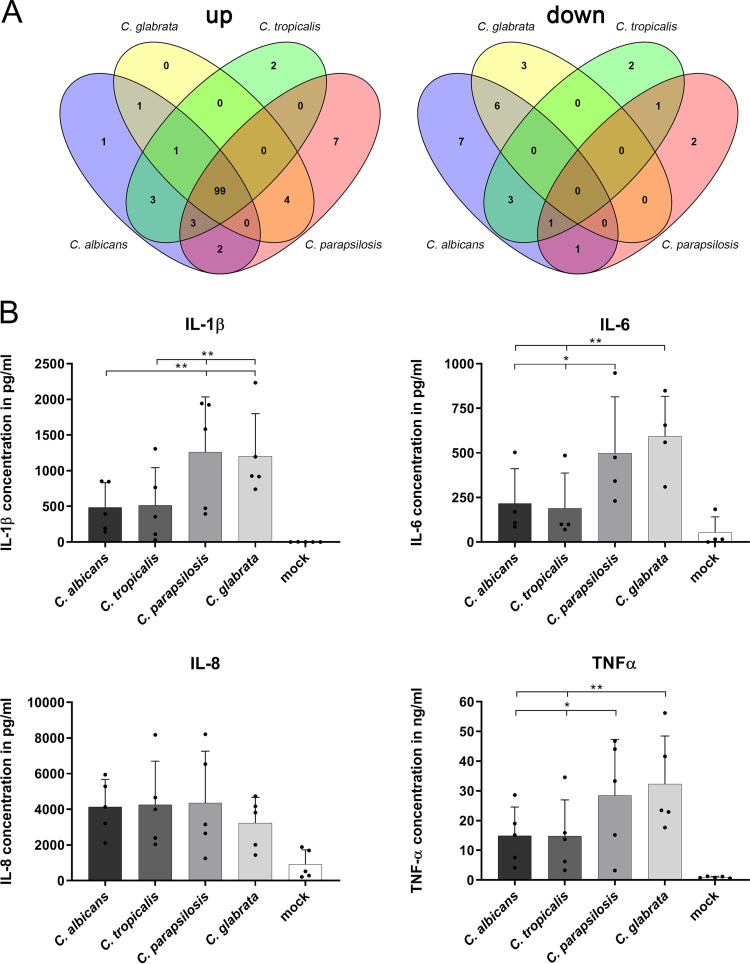
Immune system processes govern the human core response of up- and downregulated genes, and proinflammatory cytokines and chemokines are released upon *Candida* blood infection. (A) Overlap of immunological pathways from the Pathway Interaction Database (PID) with statistically significant enrichment (multiple-testing corrected *P* < 0.05). Sets of genes up- or downregulated at any time point during infection were tested. The vast majority of upregulated pathways were found in response to all infecting species; comparatively few pathways were found to be significantly downregulated. (B) Plasma levels of the proinflammatory cytokines IL-1β, IL-6, and TNF-α and the chemokine IL-8 were increased at 240 mpi compared to mock infection. C. parapsilosis and C. glabrata caused higher levels of the proinflammatory cytokines than C. albicans and C. tropicalis infections. IL-8 plasma levels were lower upon C. glabrata infection than upon infection with the other three *Candida* species. Data represent means of results from at least three (B) independent experiments performed with samples from different donors + SD (*, *P* value < 0.05; **, *P* value < 0.01; repeated measures one-way ANOVA [matched donors] controlled for false-discovery rate [FDR] at *q* = 0.05).

We went on to characterize the human core response via functional gene ontology (GO) term analyses. Genes involved in inflammatory responses, cytokine-mediated signaling, and chemotaxis were significantly upregulated 240 mpi (Fig. 2D). An analysis for enriched immune response pathways (based on the Pathway Interaction Database [50]) yielded similar results (Fig. 3A, Table S3). For all human genes upregulated during the simulated infection with any species, we found 123 significantly enriched immune pathways—99 of which were shared. These mainly comprised transcriptional activation via AP-1 and NFAT (FOS and JUN genes) but also induction of the Th17 response via interleukin-23 (IL-23) signaling, granulocyte-macrophage colony-stimulating factor (GM-CSF)-mediated macrophage differentiation, and a broad range of interleukin signaling pathways. Among the few specific responses, we observed higher levels of IL-12A and IL-12B gene expression and IL-12-dependent regulation upon infection with C. glabrata and C. parapsilosis, in agreement with previous data showing a lack of or even active repression of IL-12 production by C. albicans (51, 52) and a higher level of IL-12 release during whole-blood infection with C. glabrata than with C. albicans (53). There were 26 enriched downregulated pathways, especially at late time points, with little overlap among the species and no clear overall pattern. However, in apparent contradiction to the overall proinflammatory response, genes associated with immune processes such as endocytosis and Toll-like receptor signaling were downregulated 240 mpi (Table S4). This likely indicates a shift from pro- to anti-inflammatory processes after most fungal cells had been killed, to dampen the immune response and protect the host (54, 55).

We investigated immunoregulatory genes of the human core response in detail (Fig. S2). Again, the majority of these genes were uniformly regulated, including an immediate upregulation (up to 2,000-fold) of major proinflammatory cytokine- and chemokine-encoding genes such as *IL1B*, *IL-6*, *CXCL8*, and *TNF*. Among the pattern recognition receptor (PRR) genes, the galectin-3 gene (*LGALS3*, recognizing β-mannan) was upregulated in response to all species. The gene coding for Toll-like receptor 2 (TLR2), critical for immune responses during candidiasis (56), was predominantly upregulated in response to C. glabrata, C. parapsilosis, and C. tropicalis (and, to a lesser degree, C. albicans). Thus, the types of human transcriptional response to early *Candida* blood infections were found to be mainly uniform.

10.1128/mBio.02435-20.2 FIG S2 *Candida* blood infections cause comprehensive regulation of the immune system. A multitude of immunomodulatory genes of the human common core response were similarly upregulated (red) or downregulated (blue) in response to *Candida* blood infections 240 mpi, with *IL1A* the human gene most strongly upregulated (almost 100,000 times upon C. parapsilosis infection). See Table S2 for specific values. Download FIG S2, TIF file, 0.3 MB. Copyright © 2020 Kämmer et al. 2020 Kämmer et al. This content is distributed under the terms of the Creative Commons Attribution 4.0 International license.

As we had detected an upregulation of major proinflammatory cytokine-encoding genes, we measured plasma cytokine levels at 240 mpi. Proinflammatory IL-1β, IL-6, and tumor necrosis factor alpha (TNF-α) levels were markedly increased upon any infection compared to mock control and were higher during C. glabrata or C. parapsilosis infections (Fig. 3B). Since this triad of cytokines is released mainly by monocytes (57), this may reflect their higher rates of association with C. glabrata and C. parapsilosis. Of note, C. glabrata induced lower plasma levels of IL-8, a potent chemoattractant for neutrophils, than the other species (Fig. 3B).

In summary, similar levels of immune system activation by different *Candida* species were detected on several levels in the whole-blood infection model. Neutrophils and monocytes were activated and associated rapidly with *Candida* cells, which were efficiently killed during the course of infection. This was accompanied by a predominantly uniform response on the transcriptional level, governed by processes of the innate immune system.

### The few commonly regulated fungal pathways are highly conserved.

**FIG 4:**
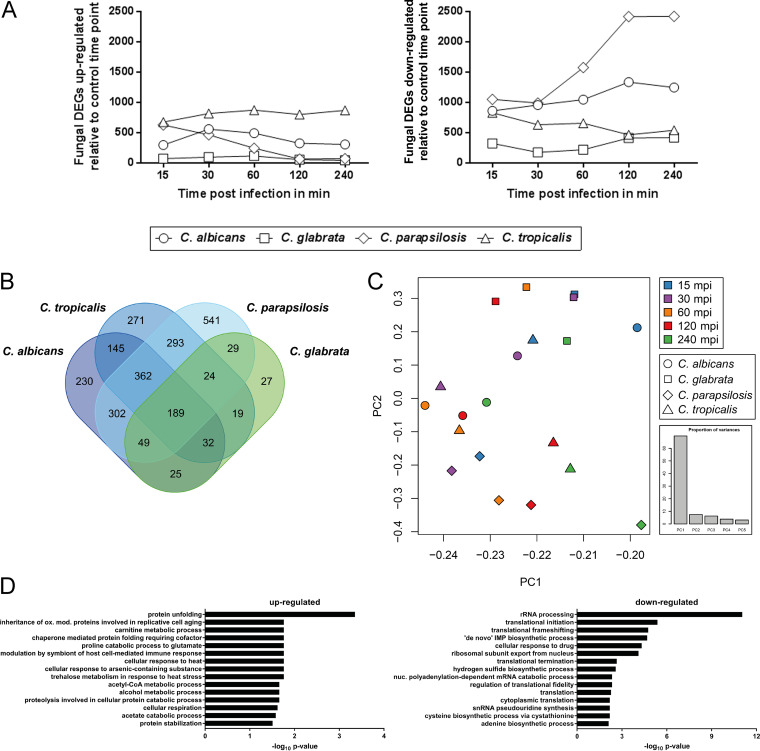
Species-specific responses govern fungal transcriptomes upon blood infection. (A) *Candida* genes were regulated with different kinetics in response to blood infection. With the exception of C. glabrata, substantial subsets of the fungal genomes were immediately regulated. Furthermore, the proportions of upregulated (left graph) and downregulated (right graph) genes differed among the four species. (B) Venn diagram showing that only a minority (189) of the orthologs are commonly regulated in response to *Candida* blood infection. (C) PCA of the fungal core response revealed no clear similarity between samples from one time point (same color) or one species (same icon). (D) Enriched categories of the common fungal core response comprised, among others, the upregulation of the unfolded protein response and the downregulation of several translational processes. CoA, coenzyme A.

Although the majority of *Candida* cells were killed in the blood environment, a considerable population of each species survived for some time, and a subpopulation was still alive at the end of the experiment. In each case, this may represent a critical mass for life-threatening bloodstream infections and transition into the organs. Therefore, transcriptional profiles of *Candida* cells exposed to blood should reflect activities which permit transient or even permanent survival in each population. We observed a reduction in fungal RNA yield and quality over the course of the infection, indicating that RNA from dead and dying fungi was quickly degraded by the ever-present human RNAses. Therefore, levels of RNA of fungal cells which realized transcriptomes beneficial for survival were enriched in our analysis of C. albicans, C. glabrata, C. parapsilosis, and C. tropicalis during blood infection. We expected that fungal cells would use comparable survival strategies in blood. Surprisingly, we instead found significant differences. In contrast to their host, *Candida* species, except C. glabrata, already showed regulation of a significant fraction of their transcriptome at 15 mpi (Table S5). This response was robust during the whole course of infection. For C. albicans, C. tropicalis, and C. parapsilosis, 35.2% (2,402 of 6,815 genes), 35.7% (2,236 of 6,258), and 47.3% (2,758 of 5,837) of their genetic repertoire, respectively, was differently regulated compared to the preculture at one time point at least. In stark contrast, only 10.5% of C. glabrata genes (552 of 5,281) were differentially expressed at a significant level at any time point during infection. Moreover, the direction of regulation differed significantly. Only C. tropicalis upregulated most of its transcripts, while the majority of genes were downregulated in all other fungi (Fig. 4A).

Although the genomes of the four *Candida* species share among them more than 3,500 orthologs, only 189 of these were commonly regulated (Fig. 4B) at any time. The transcriptional variance of this fungal core response was determined neither by the time point postinfection nor by the species, as indicated by PCA (Fig. 4C). We characterized this conserved regulation by GO term analyses (Fig. 4D) for which we assigned orthologs the same GO terms to cover ≈83% (7,184 of 8,670) of the ortholog groups and species-specific genes in our set. A key feature of the fungal core response is an extensive shutdown of protein biosynthesis and related processes such as rRNA processing, translation initiation, and purine biosynthesis (all of the corresponding genes are listed in Table S5). Glycolytic genes *ENO1*, *HXK2*, and *PFK1* were likewise universally downregulated, as were genes associated with fatty acid synthesis (*FAS1*, *FAS2*), indicating a metabolic rearrangement. In contrast, genes of the general stress response, e.g., those coding for heat shock proteins (*HSP78*, *HSP104*), were commonly upregulated. All four species also showed increased expression of genes encoding hydrolytic enzymes such as extracellular proteases, which have been linked to *Candida* pathogenicity (58–60). We consider these common responses to represent an evolutionary trait which preceded and likely enabled development of pathogenicity in different *Candida* species.

### *Candida* species pursue custom-tailored strategies to survive in blood.

**FIG 5:**
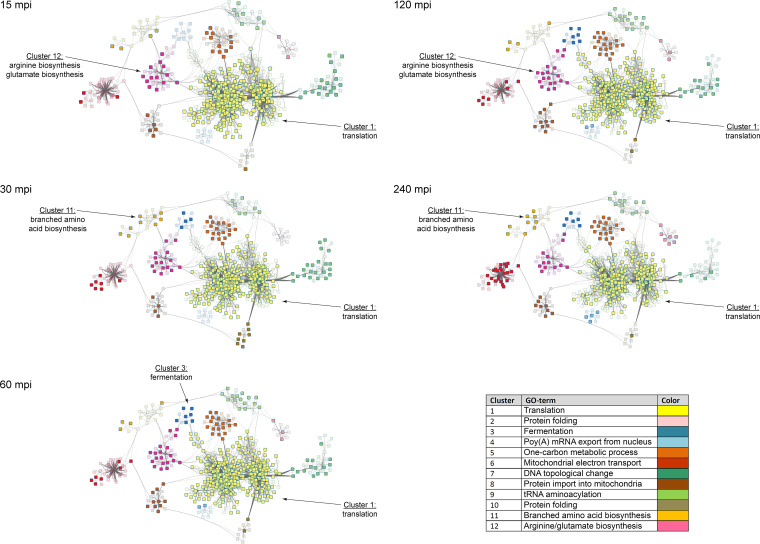
Transcriptional regulation of genes within clusters of a regulatory module is a highly dynamic process. C. albicans DEGs were used to generate a protein-protein interaction network-based regulatory module containing sets of coexpressed genes sharing a common function. Color-coded clusters within the regulatory module contained strongly connected network components which were significantly associated with distinct biological processes (Table S6), e.g., for cluster 12, arginine/glutamate biosynthesis. The regulation of each single gene is indicated by intense (differentially expressed) or transparent (not differentially expressed) coloring, according to the corresponding time point. The regulation of most of the clusters was highly dynamic during the infection process. Figure 5 is also provided as an animated image in Fig. S3 in the supplemental material.

We characterized these surprisingly individual fungal responses in more detail to determine whether survival strategies differed significantly between the species—which would indicate independent evolutionary adaptations. Using the well-annotated C. albicans genome as a reference, we generated a regulatory module of the fungal response kinetics, comprised of sets of coexpressed genes sharing a common function (61). Via GO term analyses of clusters within the regulatory module containing strongly connected network components (Fig. 5; see also Table S6), we characterized the response of C. albicans to the host over time. With this template, we analyzed the responses of all species based on orthologs.

10.1128/mBio.02435-20.3 FIG S3 Animated version of Fig. 5. Download FIG S3, GIF file, 1.6 MB. Copyright © 2020 Kämmer et al. 2020 Kämmer et al. This content is distributed under the terms of the Creative Commons Attribution 4.0 International license.

A hallmark of the C. albicans response was an immediate (15 to 30 min) and stable upregulation of the glyoxylate cycle (*ICL1*, *MLS1*) and fermentative energy production (*ADH2*, *ALD6*) (Fig. 5, cluster 3), indicating fast glucose restriction and alternative carbon source utilization. C. tropicalis and C. parapsilosis responded similarly and, furthermore, strongly upregulated genes involved in β-oxidation (*POX1-3*, *PXP2*, *FOX2*, *FOX3*, *POT1*, and *ECI1*, Table S5). However, C. parapsilosis significantly downregulated the glyoxylate cycle later, and strikingly, C. glabrata did not react with early nutrient acquisition but instead downregulated transporters for carbohydrates, amino acids, and ammonium.

**FIG 6:**
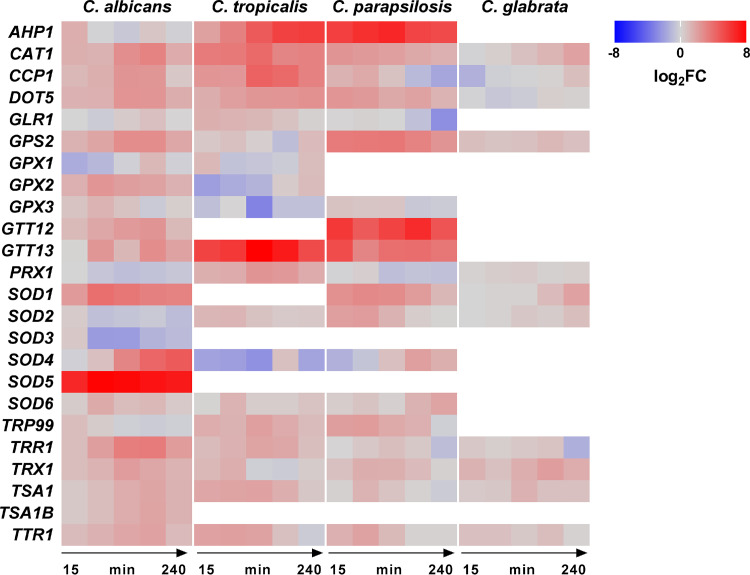
*Candida* species regulate species-specific subsets of genes involved in oxidative stress response. C. albicans, C. tropicalis and C. parapsilosis upregulated several genes associated with oxidative stress response (as determined via orthologous gene annotations from the *Candida* Genome Database), while C. glabrata did not. Genes without an ortholog are shown in white.

Engulfment by phagocytes exposes fungi to reactive oxygen species (ROS), against which *Candida* species employ a variety of generally conserved detoxifying enzymes. However, we found unique patterns in the regulation of these genes (Fig. 6A). While C. albicans strongly expressed the superoxide dismutase genes, in particular, *SOD5*, C. parapsilosis and C. tropicalis upregulated alkyl hydroperoxide reductase (*AHP1*) and putative glutathione *S*-transferase (*GTT12*, *GTT13*) genes. In contrast, C. glabrata exhibited a very restrained response, with negligible upregulation of catalase gene *CTA1* (240 mpi; log_2_ fold change [log_2_FC], 1.09). Evidently, *Candida* species evolved very different responses to ROS during blood infections.

Finally, adhesion to endothelia is essential for escape from the bloodstream. Large families of adhesin genes are found in the genomes of all investigated *Candida* species, and several of them were regulated during blood infection. C. albicans upregulated adhesins with gene-specific kinetics. While *ALS1* and *HWP2* expression decreased over time, the high transcriptional levels of *HWP1* and *ALS3* remained almost stable. Although *HWP1* was the second most highly upregulated C. albicans gene (30 mpi; log_2_FC, 12.6), its orthologs either were not regulated or were even downregulated in C. tropicalis or C. parapsilosis, respectively. Remarkably, of the 67 genes predicted for adhesin-like proteins in C. glabrata (62) only *EPA6*, *EPA7*, and *PWP1* were immediately upregulated.

### Deletions of orthologs with different regulation patterns lead to different outcomes.

On the basis of these differences in regulation of orthologous genes, we investigated the effect of their deletion on fungal survival in blood, using previously confirmed mutants of the genetically tractable yeasts C. albicans and C. glabrata (Table 1).

**TABLE 1:** Strains used in the study

Species	Name	Description^*a*^	Internal ID	Reference
C. albicans	Wild type	C. albicans WT strain SC5314	C55	105
C. glabrata	Wild type	C. glabrata WT strain ATCC 2001	C94	106
C. parapsilosis	Wild type	C. parapsilosis WT strain GA1	C118	114
C. tropicalis	Wild type	C. tropicalis WT strain DSM 4959	C30	107
C. albicans	*cph1*ΔΔ *efg1*ΔΔ	SC5314, *cph1*::FRT1/*cph1*::FRT1 *efg1*::FRT/*efg1*::FRT	M2188	108
	Reference for mutant *ras1*ΔΔ	SC5314 [CAI-4], *ura3*::imm434/*ura3*::imm434, *rps1*::*URA3*	M1202	109
	*ras1*ΔΔ	SC5314 [CAI-4], *ura3*::imm434/*ura3*::imm434, *ras1*::hisG/*ras1*::hisG *rps1*::*URA3*	M2374	110 (AH81 with CIp10)
	Reference for mutant screen	SC5314 [BWP17], *rps1*::(*HIS1 ARG4 URA3*)	M1477	111
	*bcr1*ΔΔ	SC5314 [BWP17], *bcr1*::*ARG4*/*bcr1*::*URA3 his1*::*hisG*/*his1*::*HIS1*	M1325	112
	*dur1,2*ΔΔ	SC5314 [BWP17], *dur1,2*::*ARG4*/*dur1,2*::*HIS1 rps1*::*HIS1*	M2672	
	*tec1*ΔΔ	SC5314 [BWP17], *tec1*::*ARG4*/*tec1*::*URA3 his1*::hisG/*his1*::HIS1	M1328	112
	*cat1*ΔΔ	SC5314, *ura3*::imm434/*ura3*::imm434 *his1*::hisG/*his1*::hisG *cat1*::*URA3*/*cat1*::*HIS1*	M1929	113
	*sod5*ΔΔ	SC5314, Δ*sod5*::hisG/Δ*sod5*::hisG *rps1*::*URA3*	M133	23
C. glabrata	Reference for mutant screen	ATCC 2001, *trp1*::FRT *his3*::FRT *leu2*::FRT	G38	85
	*ras1*Δ	ATCC 2001, *trp1*::FRT *his3*::FRT *leu2*::FRT CAGL0B04521::NAT1	G40-3E9	85
	*bcr1*Δ	ATCC 2001, *trp1*::FRT *his3*::FRT *leu2*::FRT CAGL0L00583g::NAT1	G40-4A5	85
	*dur1,2*Δ	ATCC 2001, *trp1*::FRT *his3*::FRT *leu2*::FRT CAGL0M05533g::NAT1	G40-4B5	85
	*tec1*Δ	ATCC 2001, *trp1*::FRT *his3*::FRT *leu2*::FRT CAGL0F04081g::NAT1	G40-1E4	85
	*cta1*Δ	ATCC 2001, *trp1*::FRT *his3*::FRT *leu2*::FRT CAGL0K10868g::NAT1	G40-2C12	85

a WT, wild type.

**FIG 7:**
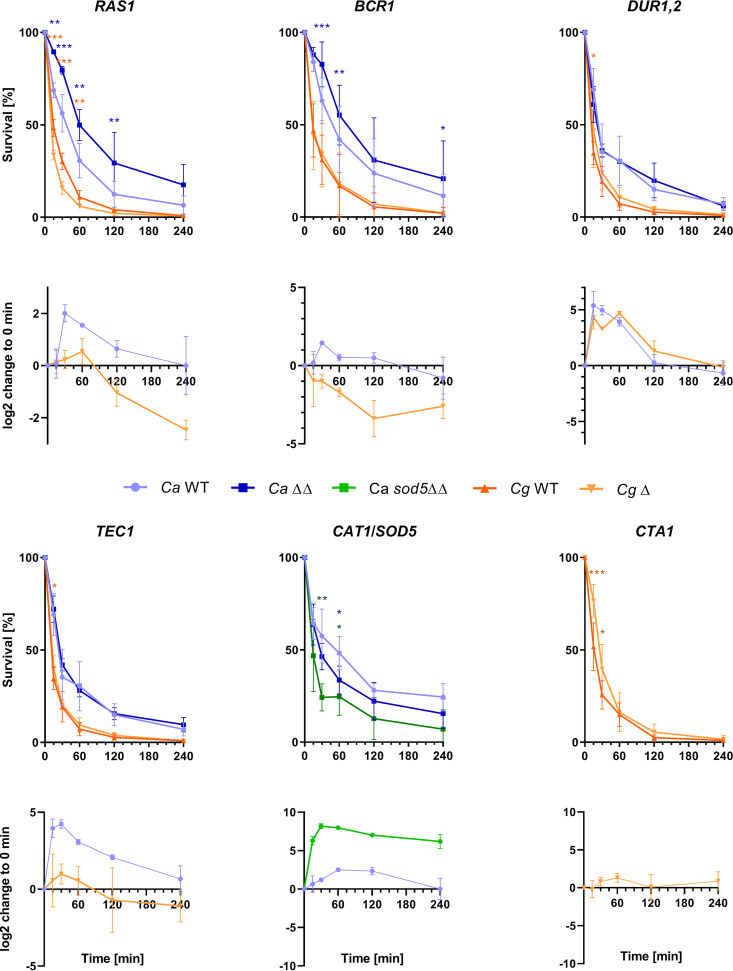
Regulation of selected genes in C. albicans and C. glabrata and effect of their deletion on survival in human whole blood. Orthologous genes with similar or different transcription profiles (lower graphs) were selected for survival tests in comparison to the corresponding deletion mutants (upper graphs; blue, C. albicans wild type [WT] or homozygous deletion mutants [ΔΔ]; orange, C. glabrata wild type or deletion mutant [Δ]; green, C. albicans *sod5*ΔΔ deletion mutant for comparison). For some genes (e.g., *SOD5*), upregulation in blood predicted a role in survival, but several mutants (e.g., C. albicans *ras1*ΔΔ) exhibited survival rates that were even increased (see main text for details). Survival experiments were performed in triplicate with samples from different donors, with means ± SD shown; repeated-measures two-way ANOVA [matched donors]; Sidak’s multiple testing-corrected *P* values, *, *P* value < 0.05; **, *P* value < 0.01; ***, *P* value < 0.001.

Ras1 is a signal transduction GTPase with established roles in morphogenesis and virulence of C. albicans (63). Interestingly, its orthologs are downregulated in all non-*albicans Candida* species, and accordingly, deletion of *RAS1* in C. glabrata reduced initial survival in blood only slightly (Fig. 7). The C. albicans gene was upregulated starting at 30 mpi, fitting its supposed functions. Surprisingly, its deletion led to a significant increase in survival of C. albicans at all time points except 4 h (the levels seen at 4 h were higher, but the difference lacked statistical significance). A similar effect was observed for the transcription factor gene *BCR1*, whose transcription was (transiently) upregulated in C. albicans but was significantly downregulated during blood infection in C. glabrata (Table S5) and in C. parapsilosis. While the corresponding C. glabrata deletion mutant was killed at a rate identical to that seen with the wild-type strain in blood, the survival rate of the C. albicans deletion mutant was again significantly higher (at 30, 60, and 240 mpi) (Fig. 7).

On the effector side, *DUR1*,*2* encodes a urea amidolyase with a role in morphogenesis of C. albicans (64). Its transcription levels follow similar patterns in C. albicans, C. glabrata, and C. tropicalis, with a steep early increase followed by a return to the base level at around 120 mpi. Despite its rapid upregulation (and in contrast to *RAS1*), deletion of *DUR1*,*2* did not appreciably affect survival of C. albicans or C. glabrata.

The transcription factor gene *TEC1* followed a pattern comparable to that seen with *RAS1*, in that its transcription was immediately increased only in C. albicans. Despite its role in murine and *Galleria* virulence of C. albicans (65, 66), survival in blood was not affected by its deletion in C. albicans or in C. glabrata.

We next focused on genes with presumed roles in survival. The strong oxidative stress response of C. albicans is reflected by significantly reduced survival of the *cat1*ΔΔ mutant and especially the *sod5*ΔΔ mutant (Fig. 7), and both genes were immediately and strongly upregulated in the wild type. C. glabrata lacked similarly strong upregulation of *CTA1*, and the corresponding mutant even showed increased early survival in blood (Fig. 7). Overall, regulation differences of orthologs frequently translate to different rates of mutant survival in *Candida* species, but the direction of this effect depends on their functional role.

## DISCUSSION

We aimed to investigate the strategies employed by different pathogenic *Candida* spp. in disseminating infection and to determine whether these represented evolutionary conservation or analogous patterns of evolution from common ancestral adaptations or completely independent evolutions. To this end, we applied an *ex vivo* whole-blood infection model to simulate a key step of blood-borne dissemination.

Upon entering the bloodstream, *Candida* cells face a new and hostile environment. Nutrients are restricted, and, most importantly, the host immune system combats the invaders. In this study, we used a whole-blood infection model developed and refined in our laboratories (23, 24) for a global comparative transcriptional analysis of the four most common pathogenic *Candida* species. Data on immune cell interactions, cytokine release, fungal survival rates, and kinetics of the host and *Candida* species transcriptional responses obtained in this study revealed an unexpected level of unique regulation on the fungal side facing a mostly uniform host response for the first 4 h of infection.

Our *ex vivo* findings corroborated earlier *in vitro* and *in vivo* studies showing that C. glabrata attracted monocytes more strongly and was more efficiently phagocytosed than C. albicans (45, 47). Similarly, higher rates of macrophage migration toward C. parapsilosis and of intracellular replication of C. parapsilosis were shown (67). It has been suggested that survival within monocytes is a fungus-driven mechanism employed by C. glabrata (25, 58, 68, 69) and C. parapsilosis (67) to evade immune surveillance. This would require an early high rate of association with blood monocytes, a conjecture which is supported by our *ex vivo* data. However, despite these observations, C. glabrata and C. parapsilosis were killed as efficiently as C. albicans and C. tropicalis. Thus, extracellular killing might be most important at least within the first 4 h of blood incubation.

Infection of human whole blood with any *Candida* species led to the release of IL-8 and proinflammatory cytokines (IL-1β, IL-6, TNF-α). This triad of cytokines is mainly produced by blood monocytes and is crucial for driving the acute-phase response to pathogens (57). C. glabrata and C. parapsilosis induced higher levels of IL-1β, IL-6, and TNF-α, suggesting stronger activation of monocytes, in agreement with their higher association rates. Recent work showed that IL-12 is a key mediator of monocyte-derived cytokine release in response to C. glabrata during whole-blood infection (53). The higher level of release of the neutrophil attractant IL-8 (also seen in previous work [45]) is in agreement with our observed frequent association of C. albicans with neutrophils.

The host responded slowly and with steadily increasing transcriptional changes to all *Candida* infections. With very few exceptions, this response was time dependent rather than species dependent. In the short term at least, the transcriptional immune reaction is thus largely independent of the infecting *Candida* species. Proinflammatory cytokine genes such as *IL-6* and *TNF* and chemokine genes such as *CCL20* were among those most highly upregulated. A previous study found the same genes upregulated with species as diverse as C. albicans, Aspergillus fumigatus, Escherichia coli, and Staphylococcus aureus (44). Genes that were fungus specific in that study, such as *FOSB* and *TBC1D7*, were similarly regulated in our experiment. This supports the idea of their potential role as general immune response markers for fungal infections. On the host side, our *ex vivo* whole-blood infection model therefore mimics vital characteristics of an early *Candida* bloodstream infection. Rapid association of immune with fungal cells triggers efficient *Candida* killing and proinflammatory cytokine release, which does not require immediate and major changes in the transcriptional response (24). Most importantly, we found that the restrained transcriptional response to different *Candida* species followed a uniform short-term program—despite measurable differences in physical immune cell interactions and severe divergence in the fungal transcriptome kinetics.

Fradin et al. were the first to interrogate the fungal transcriptional response to human whole blood in a C. albicans infection (42). With the refined whole-blood infection model (24), we looked beyond C. albicans to determine whether *Candida* species follow evolutionary conserved strategies or different strategies to survive in blood. We consider the upregulation of extracellular hydrolytic enzymes and the general heat shock response to be representative of an evolutionary older response preceding and enabling the individual pathogenicity programs. The translational shutdown likely represents a response to the nutrient limitation in blood and corroborates earlier studies of C. albicans blood (23) and macrophage (70, 71) infections and of C. glabrata infection of macrophages (58) as well as of A. fumigatus blood infection (72). This indicates that early downregulation of translation is a common principle in *Candida* or even fungal pathogenesis. The transcriptional response of C. glabrata seemed to be much less pronounced than that of other species. It is possible that this fungus is better at storing the nutrients necessary for survival for 4 h in our setup. However, in our ongoing (unpublished) work, we have found similarly restrained transcriptional responses in epithelial infections also, indicating that such responses may represent a general principle in C. glabrata-host interactions.

Interestingly, most of the fungal transcriptional regulations were dissimilar between or even unique to one of the *Candida* species. This concerns almost all aspects of fungal adaptation to the host, from the use of alternative energy sources to pathogenicity mechanisms. For instance, while glycolytic enzymes were commonly downregulated, the alternative glyoxylate cycle was upregulated in three of the four species, but not in C. glabrata—although it has been suggested as a potential drug target due to its ubiquitous upregulation in microbial infections (73–77). For some fungi, fatty acids may serve as energy and carbon sources during infection, as indicated by the upregulation of genes for β-oxidation, lipases, and carnitine transport most prominently by C. parapsilosis and less so by C. tropicalis. This carbon metabolic response was quite distinct from that seen with C. albicans, which lacked strong induction of fatty acid catabolism, and especially from that seen with C. glabrata.

The expression of adhesins enables attachment to the blood vessel endothelium. In agreement with previous findings, we found strong and rapid induction of adhesin gene families in C. albicans (42) known to be involved in endothelial cell adherence (reviewed in references 78 and 79). Among the members of the gene families in C. albicans, C. tropicalis, and C. parapsilosis, we found clearly different expression patterns, in accordance with the high genetic variability of the *ALS* and *IFF*/*HYR* gene families (80). The C. glabrata genome contains a repertoire of unrelated adhesion-mediating *EPA* genes (62, 79). Indeed, *EPA6* and *EPA7*, known to mediate adherence to endothelial cells (81), were upregulated early in our model. Evidently, each *Candida* species follows the same strategy of adhesion but acquired its adhesion capability to host cells independently.

The presence of such diverse solutions to adapt to similar host environments led us to conclude that, based on a common core response, the individual realizations of pathogenesis evolved mostly independently in the four *Candida* species and did not necessarily follow the same evolutionary trajectories. This assumption was supported by the different effects of deletions of orthologous genes in C. glabrata and C. albicans on survival. Deletion of genes such as *DUR1*,*2* had no effect despite their immediate, albeit transient, cross-species upregulation; they may not be relevant for short-term survival. Other genes, like *CAT1*/*CTA1*, showed different regulation patterns which somewhat corresponded to their importance for blood survival. In fact, the transcription and the contrasting effects of the catalase gene deletions in C. glabrata and C. albicans matched their roles in animal infection models—C. glabrata *CTA1* is dispensable in murine infections (82) (and human blood), while C. albicans *CAT1* is required (83), as are other ROS-detoxifying genes such as *SOD5*. Why the lack of *CTA1* in C. glabrata seems to very slightly increase survival is not immediately clear; however, a catalase-negative strain of Saccharomyces cerevisiae was shown previously to exhibit increased resistance to nitrosative stress (84). Potentially, C. glabrata benefits from such resistance in its interaction with blood phagocytes.

Deletion of *RAS1*, downregulated in all species but C. albicans, is known to slow growth of C. glabrata in minimal media (85), but growth was likely not a relevant factor in our experiment. As expected, its deletion had no discernible effect on C. glabrata survival. The increased survival of the C. albicans deletion mutant seems counterintuitive, as its *RAS1* gene is (uniquely) upregulated mid-infection. However, unexpected, complete survival of such a mutant in neutrophil phagosomes was observed very recently (86)—fitting our observation of a strong association of C. albicans with neutrophils. Those authors speculated that downregulation of *RAS1* would enable survival of C. albicans within immune cells (86). Our data suggest instead that its upregulation by C. albicans may be detrimental in blood, while other *Candida* species might benefit from its downregulation.

How can these observations be reconciled with the success of C. albicans as a pathogen? Some models of *Candida* pathogenicity posit that while C. albicans relies on fast escape from the bloodstream and immune effector cells, C. glabrata may remain within monocytes to avoid immune surveillance and disseminate (87, 88)—a conjecture which is also supported by the higher monocyte association rates observed in our experiments. Thus, C. albicans likely both responds to the immediate threat (oxidative stress, e.g., *SOD5*) and, preemptively, upregulates genes which enable bloodstream escape and tissue invasion (e.g., *RAS1*). Our nonfilamentous mutant with deletion of *EFG1* and *CPH1*, which also lacks upregulation of hypha-associated genes such as *SOD5*, showed reduced survival. Thus, the immediate serum-induced formation of hyphae and their associated transcriptional program seem to enable C. albicans to survive better than the other *Candida* species in human blood. Our findings also suggest that the survival of fungal cells in the complex blood environment is probably not reliant on individual gene regulations but instead represents the sum of several partly redundant or overlapping responses. Otherwise, the deletion of single highly regulated genes would have had much more prominent effects on survival than we have observed.

This finding can be considered an interesting example of adaptive prediction (89), in which the pathogen upregulates genes which have no discernible function in its current environment for later gains. Overall, these findings support our notion of significant differences in the survival (and thus pathogenesis) strategies of *Candida* species. A caveat in our experiments concerns the preculture, and parts of the response could be attributed to the change in environment *per se*; however, transition into the bloodstream constitutes a significant change *in vivo* as well and elicits a fast transcriptional response in the fungus. It must also be noted that the transcriptional analysis may have included RNA of dead fungal cells. However, the human host is rich in RNAses, and we saw a steady decline in RNA yield (and quality) over time, indicating that RNA of dead fungi was quickly degraded. Thus, we expect an enrichment of survival-type transcriptomes in our data, which were obtained from still intact mRNA. A targeted transcriptional analysis of the surviving fraction alone would potentially be better suited to answer specific questions about the best survival strategy. However, this approach is limited by technical challenges, ranging from the differentiation of dead versus dying cells to the time that methods like fluorescence-activated cell sorter (FACS) analysis would require, during which the fungal transcriptome would be changed considerably by the sheer physical stress on the cells.

Taking the results together, we performed a comprehensive analysis of *Candida* blood infections and found that the human transcriptomes, governed by an innate immune system response, are largely species independent and highly similar during the early phase of infection. In stark contrast, the strategies of different *Candida* species of different levels of evolutionary relatedness differ strongly under conditions of exposure to human whole blood. As indicated by the presence of interspersed nonpathogenic species in the phylogenetic tree (10), the investigated *Candida* species have evidently independently evolved strategies to survive in the harsh blood environment. In addition, we found indications of a small common set of reactions, including stress and metabolic responses. In particular, the responses to, e.g., oxidative stress and starvation likely resulted from previous adaptations to the environment and to other hosts and, most likely (especially for C. albicans and, potentially, for C. tropicalis and C. glabrata), commensalism and now allow survival in the host and, consequently, pathogenicity.

These findings have several important consequences. For example, while it will be difficult to identify fungal gene products as general biomarkers for fungal bloodstream infections, it is likely that species-specific fungal markers and general host biomarkers for fungal infections can be identified. Our data further suggest that the use of C. albicans as the model organism for *Candida* virulence can lead to inaccurate concepts of pathogenicity. This is, for example, demonstrated by C. glabrata with its very limited transcriptional response. As all four pathogens are major causes of candidemia, our concept of fungal virulence in general, even within the *Candida* species, likely needs to change even more toward the idea of multiple virulence strategies.

## MATERIALS AND METHODS

### Ethics approval and consent to participate.

Human peripheral blood was collected from healthy volunteers with written informed consent. This study was conducted according to the principles expressed in the Declaration of Helsinki. The blood donation protocol and use of blood for this study were approved by the institutional ethics committee of the University Hospital Jena (permission number 2207-01/08).

### Strains and culture conditions.

C. albicans SC5314, C. glabrata ATCC 2001, C. tropicalis DSM 4959, and C. parapsilosis GA1 strains (Table 1) were maintained as glycerol stocks and restreaked on yeast extract-peptone-dextrose (YPD) agar plates. For experiments, single colonies were grown overnight in YPD at 30°C and reinoculated in fresh YPD at 30°C to reach mid-log phase.

### Whole-blood infection model.

Cells of the different strains were harvested in 1× PBS (phosphate-buffered saline) and diluted in an appropriate concentration. Human whole blood was freshly drawn from healthy volunteers and subjected to anticoagulation with recombinant Hirudin (Sarstedt, Nuremberg, Germany). Immediately, yeast cells were added at a concentration of 1 × 10^6^ cells per ml blood and further incubated at 37°C as indicated. For mock infection samples, 1× PBS was used.

To determine fungal survival during whole-blood infection, the initial inoculum was determined by dilution plating. After 15, 30, 60, 120, and 240 min postinfection, 10 μl of each infected blood sample was diluted in 1× PBS and immediately plated onto YPD agar plates to determine CFU counts in technical triplicate. Each strain was tested independently with blood from at least three different healthy donors.

### Flow cytometry of immune cell interaction and activation.

C. albicans, C. glabrata, C. tropicalis, and C. parapsilosis strains were grown as previously described (strains and culture conditions). Aliquots were stained with FITC (fluorescein isothiocyanate), added at a concentration of 1 × 10^6^ cells per ml blood, and incubated at 37°C as indicated. To distinguish different immune cell populations, whole blood was stained with mouse anti-human CD3-PerCP (CD3-peridinin chlorophyll protein; clone SK7, T cells), CD19-PE (CD19-phycoerythrin; clone HIB19, B cells), CD45-PE-Cy7 (clone HI30, leukocytes), CD56-V450 (clone B159, NK cells), and CD66b-PE (clone G10F5; polymorphonuclear leukocyte [PMN]) obtained from BioLegend. Monocytes were stained with mouse anti-human CD14-PerCP (clone 47-3D6) from Abcam. Stained samples were treated with FACS lysing solution (BD), washed, and acquired immediately. For raw data analysis, FlowJo v10.0.8 software was used. The presence of activation markers was determined with mouse anti-human CD11b-V450 (clone ICRF44) from BD and CD16-BV510 (clone 3G8) and CD69-APC (CD69-allophycocyanin; clone FN50) from BioLegend. Stained samples were treated with FACS lysing solution (BD), washed, and acquired immediately. For raw data analysis, FlowJo v10.0.8 software was used.

### Blood smears.

Blood smears of samples infected with C. albicans, C. glabrata, C. tropicalis, and C. parapsilosis were prepared at indicated time points and stained with May-Grünwald-Giemsa stain, dried, and microscopically visualized.

### Quantification of cytokines.

The amount of IL-1β, IL-6, IL-8, and TNF-α was determined by ELISA according to the manufacturer’s protocol (eBioscience). After 240 mpi, infected blood samples were centrifuged to obtain plasma and immediately frozen in liquid nitrogen. Cytokine levels were calculated from standard dilutions of the respective recombinant cytokines.

### RNA isolation.

At indicated time points, infected blood samples were split into aliquots for separated fungal and human RNA isolations. For mock infections, aliquots were used for human RNA isolation at 240 mpi only. To isolate human RNA, aliquots were added to a PAXgene blood RNA tube (PreAnalytiX) and processed with a PAXgene blood RNA kit (PreAnalytiX) according to the manufacturer’s protocol. For fungal RNA isolation, aliquots were added to ice-cold water, centrifuged, and immediately frozen in liquid nitrogen. The cell pellet was further processed with a RiboPure-Yeast kit (Thermo Fisher Scientific) according to the manufacturer’s protocol. RNA quantity was determined with a NanoDrop 1000 spectrophotometer (Thermo Fisher Scientific), and RNA quality was verified with an Agilent 2100 Bioanalyzer (Agilent Technologies). Fungal and human RNA samples were pooled subsequently in a quantitative ratio of 1:10. All samples were prepared in three biological replicates corresponding to independent donors at independent time points (Table S1).

10.1128/mBio.02435-20.4 TABLE S1 Statistics of dual-species RNA-sequencing. This table provides an overview of the RNA-seq statistics for all four *Candida* infections and the mock infection. Download Table S1, XLSX file, 0.02 MB. Copyright © 2020 Kämmer et al. 2020 Kämmer et al. This content is distributed under the terms of the Creative Commons Attribution 4.0 International license.

10.1128/mBio.02435-20.5 TABLE S2 Differentially expressed human genes. Human DEGs in response to C. albicans blood infection are listed in an xlsx file with their log_2_ fold changes compared to time point 0. Download Table S2, XLSX file, 0.9 MB. Copyright © 2020 Kämmer et al. 2020 Kämmer et al. This content is distributed under the terms of the Creative Commons Attribution 4.0 International license.

10.1128/mBio.02435-20.6 TABLE S3 Immune-related pathways enriched in response to *Candida* infection. Enriched immune-related pathways of the human response to *Candida* infection are listed in an xlsx file for each fungus and time point. Download Table S3, XLSX file, 0.5 MB. Copyright © 2020 Kämmer et al. 2020 Kämmer et al. This content is distributed under the terms of the Creative Commons Attribution 4.0 International license.

10.1128/mBio.02435-20.7 TABLE S4 Regulation of immunoregulatory genes of the human core response. Immune-related genes of the human core response at 240 mpi are listed in an xlsx file. Download Table S4, XLSX file, 0.02 MB. Copyright © 2020 Kämmer et al. 2020 Kämmer et al. This content is distributed under the terms of the Creative Commons Attribution 4.0 International license.

10.1128/mBio.02435-20.8 TABLE S5 Differentially expressed fungal genes. DEGs from C. albicans, C. glabrata, C. tropicalis, and C. parapsilosis in response to *Candida* blood infection are listed in an xlsx file. Download Table S5, XLSX file, 1.2 MB. Copyright © 2020 Kämmer et al. 2020 Kämmer et al. This content is distributed under the terms of the Creative Commons Attribution 4.0 International license.

10.1128/mBio.02435-20.9 TABLE S6 Clusters and cluster-associated enriched biological processes of C. albicans DEG-based regulatory module. Cluster-associated enriched biological processes of all clusters and the corresponding cluster-associated enriched biological processes are provided as an xlsx file. Download Table S6, XLSX file, 0.1 MB. Copyright © 2020 Kämmer et al. 2020 Kämmer et al. This content is distributed under the terms of the Creative Commons Attribution 4.0 International license.

### RNA sequencing.

Library preparation and RNA sequencing were carried out at GATC Biotech (Constance, Germany). After poly(A) filtering, mRNA was fragmented and cDNA libraries were generated for each sample. Single-sequence reads (50 bp) were produced using an Illumina HiSeq 2500 platform.

### RNA-seq data preprocessing.

Single-ended, 50-bp Illumina HiSeq 2500 raw reads were quality trimmed with Trimmomatic v0.32 (90). Homo sapiens genome GRCh38 and annotations were downloaded from the ENSEMBL database (91). C. albicans SC5314 assembly 22, C. glabrata CBS138, C. parapsilosis CDC317, and C. tropicalis MYA-3404 genomes and corresponding genome annotations were downloaded from the Candida Genome Database (CGD) (92). For C. albicans, polyadenylated transcriptionally active regions identified by transcriptome sequencing (RNA-seq) (93) were added to the annotation. Ortholog information was obtained from the CGD by downloading the table of orthologs (www.candidagenome.org/download/homology/orthologs/) at the time of data evaluation. All sequencing reads were mapped against concatenated genomes of H. sapiens and each of the four *Candida* species using TopHat v2.1.0 (94). Read mapping was carried out, and only uniquely aligned hits were kept for further analysis. Transcriptome coverage was calculated as mapped reads multiplied by read length and divided by transcriptome length. featureCounts v1.4.3 (95) was applied to count the number of reads within annotated genes. Human and pathogen genes were tested individually for significant differential expression. DESeq2 (96) was used to calculate adjusted *P* values based on count values. Mean reads per kilobase per million (RPKM) and log_2_FC values were calculated manually. Afterward, the following cutoffs were applied: adjusted *P* value of <0.01, abs(log_2_FC) value of ≥1.5, and RPKM value of ≥1 for at least one time point.

### Expression data analyses.

The “prcomp” function provided by the GNU R package Stats (97) was utilized to apply PCA of log_2_FC values for all host genes to any *Candida* or mock infection. The mock infection samples (240 mpi) had no dedicated counterpart at 0 mpi. We calculated four separate log_2_FC values for the comparison against 0 mpi with C. albicans, C. glabrata, C. parapsilosis, and C. tropicalis (known open reading frames plus polyadenylated transcriptionally active regions for C. albicans).

Functional Gene ontology categories enriched for DEGs were identified with FungiFun2 (96) using hypergeometric distribution and Benjamini-Hochberg-corrected *P* values of <0.05 and REVIGO (98). Ortholog information for *Candida* species was retrieved from the CGD. DEGs with orthologs in C. albicans, C. glabrata, C. parapsilosis, and C. tropicalis were quantitatively compared. DEGs of H. sapiens data sets were quantitatively compared. Similarly, pathway analyses were performed on significantly regulated genes using innateDB (99). Pathways from the PID NCI and PID BIOCARTA databases were tested for overrepresentation by the regulated human gene lists at any time point of infection (*P* value < 0.05, Benjamini-Hochberg-corrected hypergeometric distribution).

### Module.

ModuleDiscoverer was applied as described previously (61) to identify the regulatory module. For this analysis, only genes which were differentially expressed at one of the measured time points at least were considered. In addition, a high-confidence (score > 0.7) protein-protein interaction network (PPIN) of C. albicans was downloaded from STRING version 9.1 (100). Both the DEGs and the C. albicans PPIN were taken as input for ModuleDiscoverer. Identifier annotations provided by CGD (101) were used. Submodules of the resulting regulatory module with fewer than 10 network components were not considered. The clustering of the regulatory module was performed in the programming language R, version 3.4.1, using the generalized topological overlap measure regarding second-order connections as described previously (102). A cutoff of 0.65 was chosen to receive the clusters. Cytoscape version 3.2.1 (103) was used for visualizing the regulatory module. For performing GO term enrichment analyses concerning biological processes, FungiFun2 (96), including Fisher’s exact test and Benjamini-Hochberg false-discovery-rate correction, was applied to each submodule and cluster. GO terms composed of at least two members, associated with at least two components, and leading to adjusted *P* values of >0.05 were considered significantly enriched.

### Statistical analyses.

All experiments were done in at least three biological replicates with blood from nonidentical donors and independent fungal cell cultures. Data sets are reported as means ± standard deviations (SD). Statistical significance was calculated using two-way analysis of variance (ANOVA) (killing, immune cell association and activation, quantitative PCR [qPCR]) or one-way ANOVA (cytokine release) with multiple comparisons or false-discovery-rate correction. Probability values are indicated as follows: *, *P* value of <0.05; **, *P* value of <0.01; ***, *P* value of <0.005; ****, *P* value of <0.0001.

### Data availability.

The RNA-seq data set generated and analyzed during the current study has been deposited in NCBI’s Gene Expression Omnibus (104) under the GEO record GSE114180.
