# Supplementary figures and images for: Survival Strategies of Pathogenic Candida Species in Human Blood Show Independent and Specific Adaptations
Source: mBio. 2020 Oct 6;11(5):e02435-20. doi: 10.1128/mBio.02435-20 (PMC7542370; doi:10.1128/mBio.02435-20)

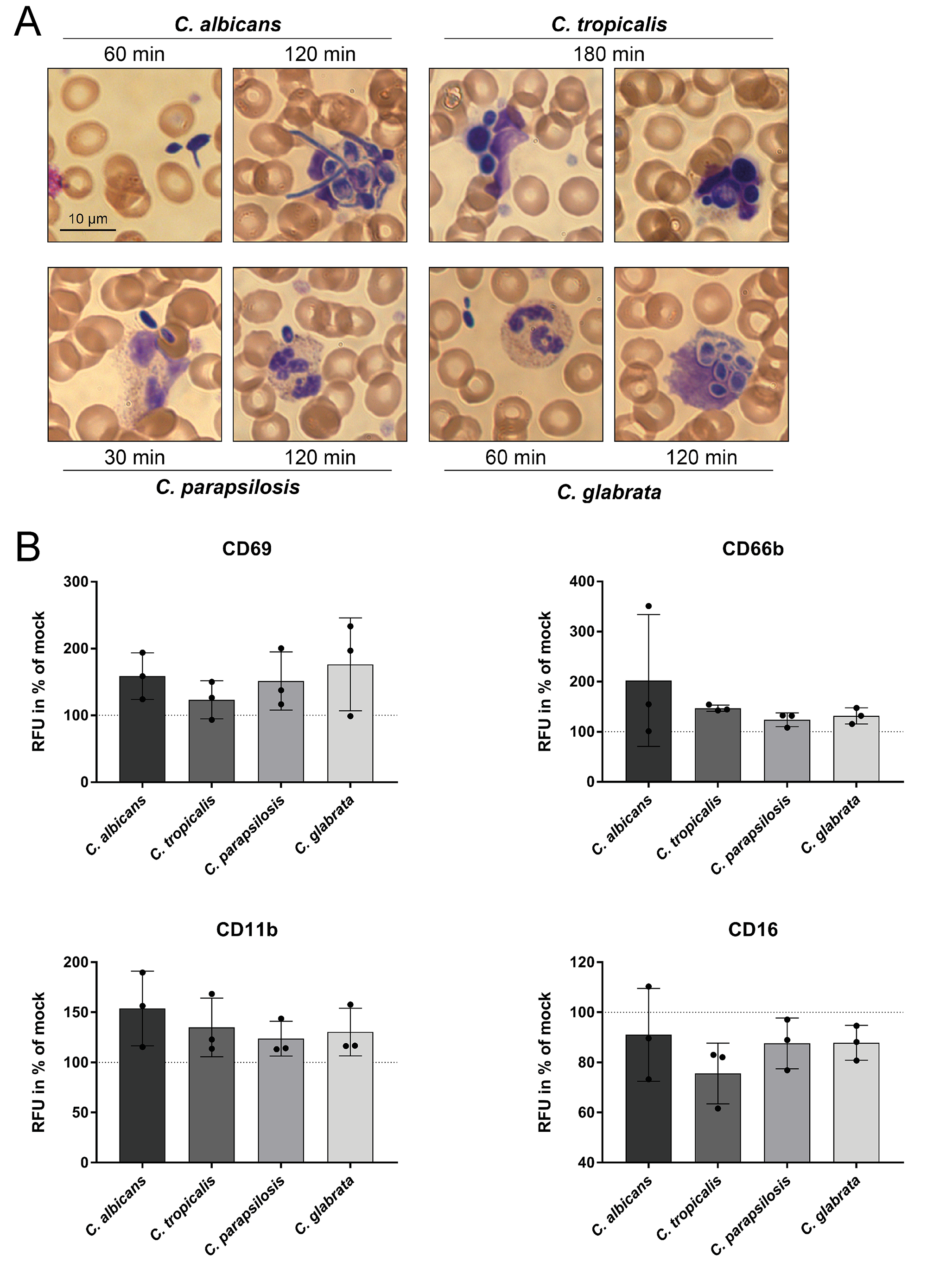

Supplement: FIG S1 [file mBio.02435-20-sf001.tif]

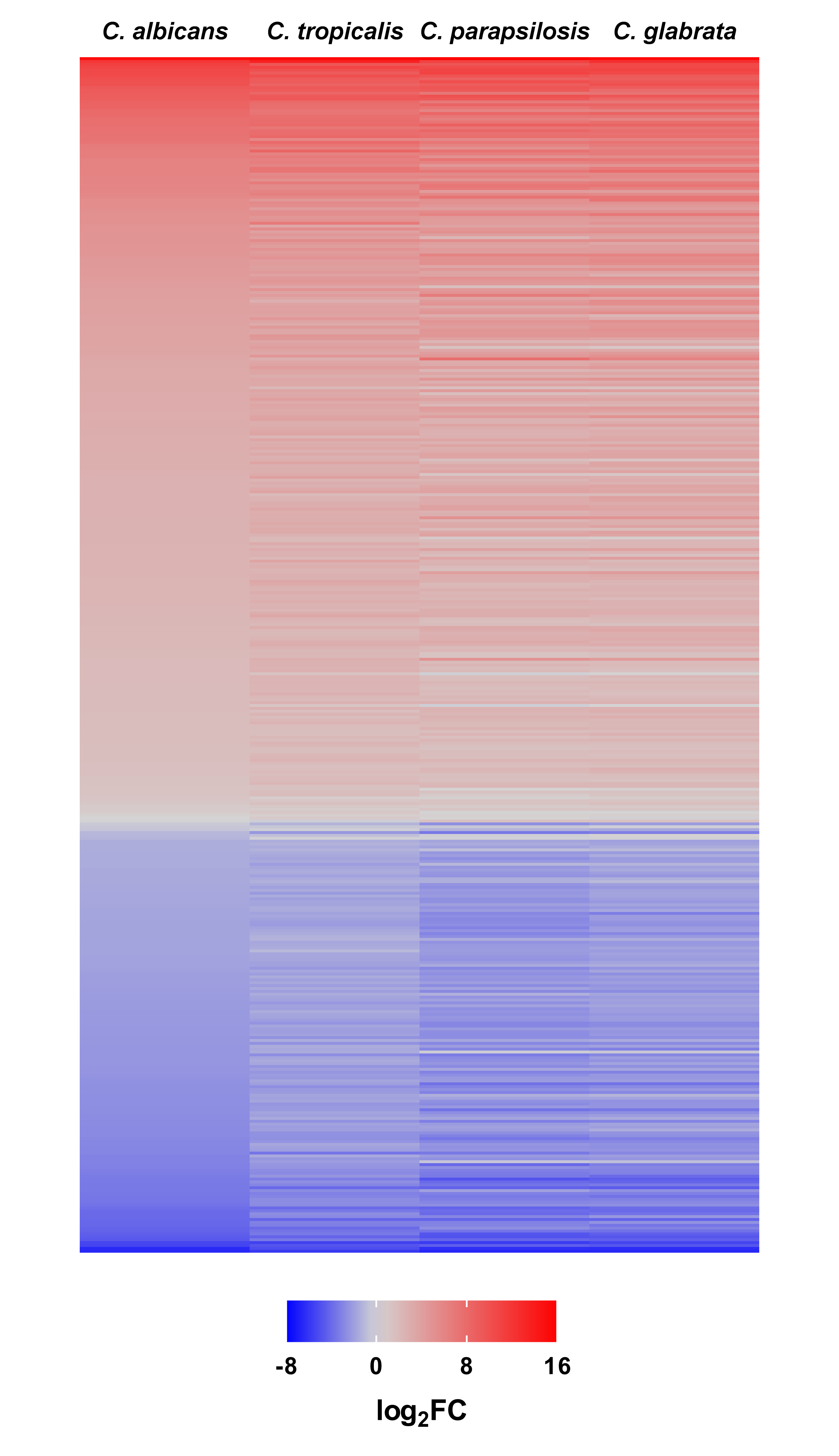

Supplement: FIG S2 [file mBio.02435-20-sf002.tif]

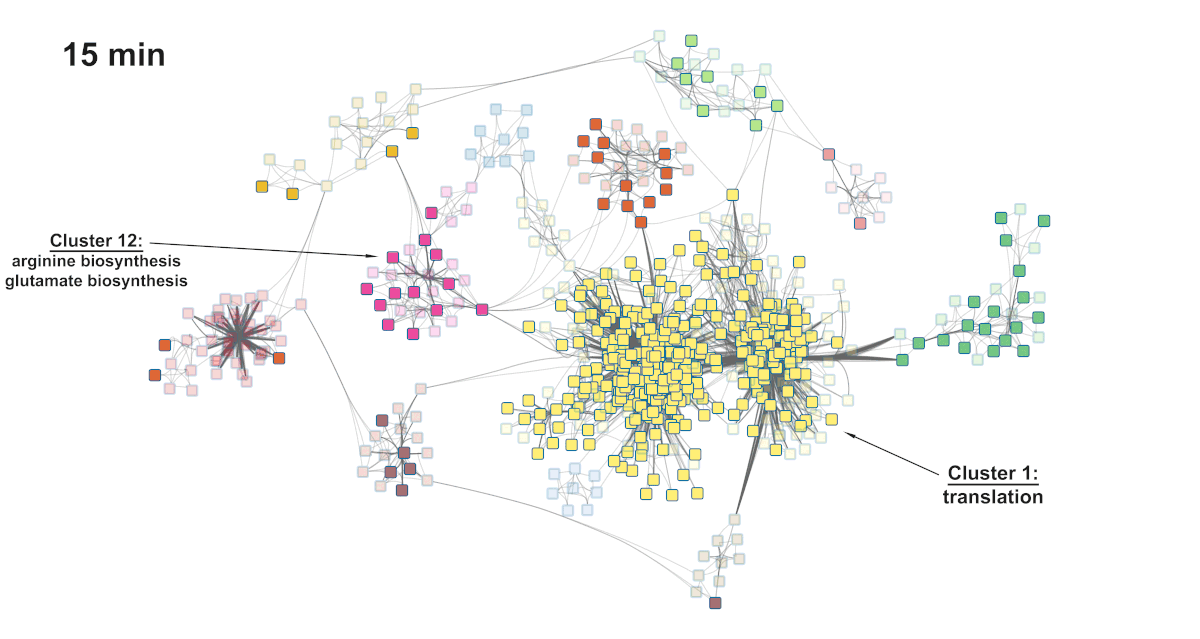

Supplement: FIG S3 [file mBio.02435-20-sf003.gif]
